# Clinical pharmacy activities in chronic kidney disease and end-stage renal disease patients: a systematic literature review

**DOI:** 10.1186/1471-2369-12-35

**Published:** 2011-07-22

**Authors:** Gunar Stemer, Rosa Lemmens-Gruber

**Affiliations:** 1 Department of Pharmacology and Toxicology, University of Vienna, Althanstraße 14, 1090 Vienna, Austria; 2 Pharmacy Department, Vienna General Hospital, Währinger Gürtel 18-20, 1090 Vienna, Austria

## Abstract

**Background:**

Chronic kidney disease (CKD) and end-stage renal disease (ESRD) represent worldwide health problems with an epidemic extent. Therefore, attention must be given to the optimisation of patient care, as gaps in the care of CKD and ESRD patients are well documented. As part of a multidisciplinary patient care strategy, clinical pharmacy services have led to improvements in patient care. The purpose of this study was to summarise the available evidence regarding the role and impact of clinical pharmacy services for these patient populations.

**Methods:**

A literature search was conducted using the *Medline*, *Embase *and *International Pharmaceutical Abstracts *databases to identify relevant studies on the impact of clinical pharmacists on CKD and ESRD patients, regarding disease-oriented and patient-oriented outcomes, and clinical pharmacist interventions on drug-related problems.

**Results:**

Among a total of 21 studies, only four (19%) were controlled trials. The majority of studies were descriptive (67%) and before-after studies (14%). Interventions comprised general clinical pharmacy services with a focus on detecting, resolving and preventing drug-related problems, clinical pharmacy services with a focus on disease management, or clinical pharmacy services with a focus on patient education in order to increase medication knowledge. Anaemia was the most common comorbidity managed by clinical pharmacists, and their involvement led to significant improvement in investigated disease-oriented outcomes, for example, haemoglobin levels. Only four of the studies (including three controlled trials) presented data on patient-oriented outcomes, for example, quality of life and length of hospitalisation. Studies investigating the number and type of clinical pharmacist interventions and physician acceptance rates reported a mean acceptance rate of 79%. The most common reported drug-related problems were incorrect dosing, the need for additional pharmacotherapy, and medical record discrepancies.

**Conclusions:**

Few high-quality trials addressing the benefit and impact of clinical pharmacy services in CKD and ESRD patients have been published. However, all available studies reported some positive impact resulting from clinical pharmacist involvement, including various investigated outcome measures that could be improved. Additional randomised controlled trials investigating patient-oriented outcomes are needed to further determine the role of clinical pharmacists and the benefits of clinical pharmacy services to CKD and ESRD patients.

## Background

Chronic kidney disease (CKD) represents a major public health problem in developed and developing countries. It is estimated that approximately 5% of the adult U.S. population is affected by CKD, which is defined as serum creatinine concentrations greater than 1.2 to 1.5 mg/dL [1]. The European Kidney Health Alliance (EKHA) reports that approximately 10% of European citizens are affected by some degree of CKD [2].

CKD and end-stage renal disease (ESRD) are associated with an increased risk of mortality, increased rate of hospitalisation, and decreased life expectancy [3]. Progression from early to late stages of CKD generally results in the onset of new symptoms and concomitant complications. Frequent complications and comorbidities of CKD include fluid and electrolyte abnormalities, anaemia, secondary hyperparathyroidism and renal osteodystrophy, hypertension and hyperlipidaemia, metabolic acidosis, and several other comorbidities involving malnutrition, pruritus and uremic bleeding. CKD patients are at increased risk of cardiovascular disease (CVD), which includes coronary heart disease (CHD), cerebrovascular disease, peripheral vascular disease, and heart failure. The management of underlying and evident comorbidities (either as causes or consequences of CKD) and the prevention or delay of its progression to ESRD are complex.

In ESRD patients, the initiation of renal replacement therapies (RRTs), such as long-term dialysis (including haemodialysis (HD) or peritoneal dialysis (PD)) or transplantation, is usually indicated to relieve uremic symptoms and detoxify, whereas kidney transplantation (cadaveric or living donor transplantation) is the therapy of choice for ESRD [4].

Multidisciplinary health care teams of physicians, nurses, dieticians, and clinical pharmacists share the goal of preventing disease progression and managing comorbid conditions in CKD and ESRD patients. As specialists in pharmacotherapy, clinical pharmacists are routinely involved in patient care and interact with other health care professionals, addressing multiple, often unmet needs for pharmacotherapy optimisation. Ideally, this happens through a preventive, rather than a reactive, approach. Evidence from the literature supports the involvement of clinical pharmacists in several disease areas and underlines the positive patient outcomes and improvement of care that result [5,6].

The medical management of predialysis and dialysis patients involves complex and highly variable pharmacotherapy, including frequent monitoring and evaluation to ensure optimal pharmacotherapy, adherence to medication, and control of comorbidities and other risk factors. A high number of prescribed medications, poor medication adherence, and frequent dosage changes may contribute to drug-related morbidity and related problems [7]. Several studies report poor quality and gaps in the care of CKD patients with respect to the treatment of comorbidities, referrals to specialists, and the preparation for RRTs [8,9].

Clinical pharmacists are directly engaged in the care of CKD and ESRD patients in different settings. Various possibilities and opportunities for clinical pharmacists to contribute to this field are described and exemplarily supported by evidence in an American College of Clinical Pharmacy (ACCP) opinion paper [10].

This literature review aims to systematically summarise the published evidence on the role of clinical pharmacists in the care of CKD and ESRD patients across different settings, to synthesise and highlight findings on the impact of clinical pharmacists, their various key activities, and their main areas of involvement, and to describe the different characteristics of clinical pharmacy services for the CKD and ESRD patient population.

## Methods

A literature search was conducted using the *Medline *(1970 - Week 46, 2010), *Embase *(1996 - Week 45, 2010) and *International Pharmaceutical Abstracts *(IPA) (1970 - Oct 2010) databases to identify relevant articles. In *Medline*, the following combinations of Medline Medical Subject Headings (Mesh) terms were used as our search strategy: ("pharmacy service, hospital" OR "pharmacists" OR "pharmaceutical services") AND ("renal insufficiency" OR "kidney" OR "renal replacement therapy"). In *Embase *and *IPA*, the search strategy combined the terms ("clinical pharmacy" OR "pharmaceutical care" OR "pharmacist" OR "hospital pharmacy") AND ("renal insufficiency" OR "kidney" OR "renal replacement therapy"). The references sections of the returned publications and review articles were further screened for additional hits. Data were extracted and reviewed by the first study author (GS) and independently reviewed by the second author (RLG). Discrepancies were solved by discussion among the study authors.

All studies addressing the impact of clinical pharmacy services (either at the patient or the physician level) on the care of CKD and ESRD patients for both HD and PD were included. Therefore all studies reporting on disease-oriented and patient-oriented outcomes, and clinical pharmacist interventions on drug-related problems (DRPs) together with the physician acceptance rate, were assessed. Studies addressing the impact of clinical pharmacy services in kidney transplantation were excluded. A detailed review of these kinds of services was recently published [11]. Detailed inclusion and exclusion criteria are described in Table 1. The weakest study design included was observational and solely descriptive, as a high number of randomised controlled trials could not be anticipated. Results published in abstract form (e.g., congress abstracts) were included only if they provided numerically assessable data, such as outcome data, the number of resolved DRPs, or physician acceptance rates.

**Table 1:** Inclusion and exclusion criteria for literature search

	Included	Excluded
**Study types**	(Randomized) controlled trials ((R)CTs), descriptive studies (DS), before-after studies (BAS) with interventional data	Case reports, case studies, surveys, cost-effectiveness studies, narrative reviews
**Interventions**	Any type of clinical pharmacist intervention embedded in comprehensive clinical pharmacy activities if data were assessable numerically and outcomes were reported	Solely screening for inappropriate renal dosing, evaluations of computerised decision support systems
**Language**	Publications in English and German	Any other language

Predefined data parameters (namely, the study design, duration and setting, the number of included patients, the types of interventions, the relevant outcomes, the results, and available statistical values) were extracted from the literature, summarised in an Excel spreadsheet, and reviewed.

## Results

**Figure 1:**
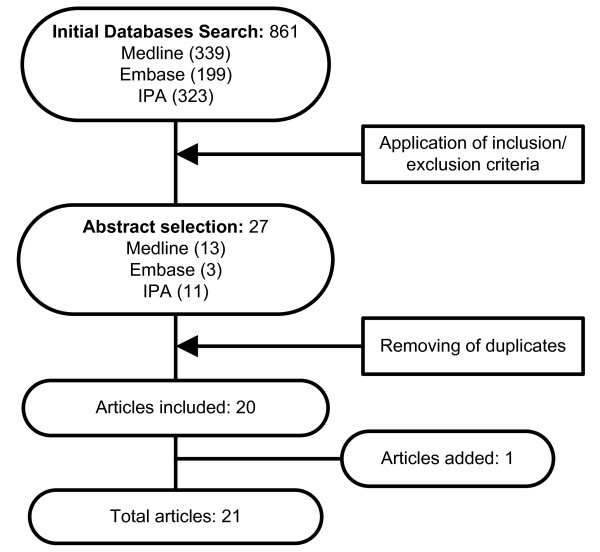
**Flowchart of search strategy and results**. IPA International Pharmaceutical Abstracts.

The initial *Medline*, *Embase *and *IPA *searches yielded 339, 199, and 323 citations, respectively. The detailed search results are described in Figure 1.

A total of 861 citations were initially screened for inclusion criteria, and after removing duplicates, a total of 21 citations remained for full review and analysis. The predominant reason for exclusion was a lack of interventional and/or assessable data. Several initial citations had to be excluded because they provided data only on the impact of screening on appropriate renal dosing, with or without computerised support, or they provided only economic data.

### General study characteristics

Detailed descriptions on the included studies of CKD and ESRD patients, including relevant interventions, outcomes, and results, are shown in Tables 2 and 3, respectively. Three study types were identified, including 14 descriptive studies (DSs) (66.7%), four (randomised) controlled (R)CTs (19%), and three before-after studies (BASs) (14.3%). A total of seven (33.3%) of the published studies were only available as abstracts. The earliest included study was published in 1993. The study sites were predominantly located in the United States (n = 16, 76.2%). The majority of the studies investigated the impact of the clinical pharmacist on the HD patient population only (n = 15, 71.4%). Six studies (28.6%) addressed care issues in CKD patients. Only two studies (9.5%) [12,13] included PD patients. Most of the studies were performed in an ambulatory HD or CKD patient care setting (n = 17, 81%), whereas only four studies contained data on in-hospital clinical pharmacist activities (see Tables 2 and 3). Using data from 18 reported studies, the median (range) number of study participants was 60 (10-408), and the median (range) study duration was six (1-32) months.

**Table 2:** Detailed description of the included publications on CKD patients

**First author**, (Year), Population	Design	**N (INT/CT)**^**a**^	Duration (months)	Interventions	**Relevant outcomes**^**b**^	Results	p-Value
Lim SB et al. [14], (2003), CKD inpts	DS	60	2	MR, therapeutic monitoring, feedback to physicians	No./Types of DRPs	86	
*Abstract*					*Transcription errors*	*44%*	
					*Renal dosage adjustments*	*10%*	
					PhAR	93%	
					Significance		
					*Somewhat significant*	*26%*	
					*Significant *	*67%*	
					*Very significant *	*4%*	
							
Patel HR et al. [15], (2005), CKD outpts	DS	119	NR	Review of medical records, evaluations of DRPs, therapeutic recommendations	No. of DRPs	381 (100%)	
*Abstract*					Types of Interventions		
					*Change of drugs*	*NR*	
					*Change of dosage*	*NR*	
					*Interval adjustments*	*NR*	
					PhAR	40.9%	
							
Allenet B et al. [31], (2007), CKD outpts	BAS	10	3	Pharmacist-managed anaemia educational programmes	Knowledge (% of right answers on a 7-item questionnaire) at baseline vs. follow-up at Month 3	80 ± 18/93 ± 10	NS
					QOL judged on a LAS (0-10) at baseline vs. Month 3		
					*Energy*	*3.3 ± 1.7/7.1 ± 1.7*	< 0.05
					*Daily activities*	*4.9 ± 2.1/7.7 ± 1.9*	< 0.05
					*General well-being*	*4.6 ± 2.2/7.5 ± 1.6*	< 0.05
							
Bucaloiu ID et al. [24], (2007), CKD outpts	DS	NR	32	Pharmacist-managed anaemia programmes compared to PCP-managed pts	Weekly erythropoietin dose	6.698/12.000 units	0.0001
*Abstract*					Time to achieve Hb goal	47.5/62.5 days	0.11
					Maintenance of Hb values in target range	69.8/43.9%	0.0001
					Maintenance of Tsat values in target range	64.8/40.4%	0.043
							
Joy MS et al. [25], (2007), CKD outpts	DS	128	28	Clinical pharmacist-managed anaemia programmes with darbopoietin	% of pts achieving Hb target compared to retrospective baseline analysis of data (before clinical implementation)	78/41%	
							
Lee J et al. [16], (2009), CKD outpts	CT	18 (9/9)	6	INT: PC CT: SOC	Disease control parameters: Change from baseline to last follow-up visit (INT/CT)		
*Abstract*					*Blood pressure*	*-6/+6.8 mmHg*	
					*HbA_1c_*	*-0.2/0%*	
					*Haemoglobin*	*1.05/-1.85 g/dL*	
					Medication adherence (pill count)	97.2/88.2%	

**BAS **before-after study, **CKD **chronic kidney disease, **CT **controlled trial, **DS **descriptive study, **DRP **drug-related problem, **Hb **haemoglobin, **HbA_1c _**glycosylated haemoglobin, **LAS **linear analogue scale, **MR **medication review, **No**. number, **NR **not reported, **NS **not significant, **PC **pharmaceutical care, **PCP **primary care physician, **PhAR **physician acceptance rate, **pts **patients, **QOL **quality of life, **SOC **standard of care, **Tsat **transferrin saturation

^a ^Number of included patients in the intervention (INT) or control (CT) group

^b ^For brevity, only the three most commonly performed interventions/drug-related problems are listed.

**Table 3:** Detailed description of the included publications on dialysis patients

**First author**, (Year), Population	Design	**N (INT/CT)**^**a**^	Duration (months)	Interventions	**Relevant outcomes**^**b**^	Results	p-Value
Tang I et al. [17], (1993), HD outpts	DS	NR	6	Therapeutic interventions provided by CP	No./Types of interventions	205 (100%)	
*Abstract*					*Drug selection*	*66 (32.2%)*	
					*Drug discontinuation*	*39 (19.0%)*	
					*Dose selection*	*50 (24.4%)*	
					Significance of interventions		
					*Preservation of major organ function*	*34.6%*	
					*Improvement in quality of care*	*62.4%*	
					PhAR	91.7%	
							
Kaplan B et al. [18], (1994), HD outpts	DS	24	NR	Focused DT review programmes	No. of recommendations/informative comments	114/85	
*Abstract*					PhAR	76% (implemented 70%)	
							
Grabe DW et al. [19], (1997), HD outpts	DS	45	1	DT reviews by CP Therapeutic recommendations	No./Types of DRPs	126 (100%)	
					*Drug interactions*	*35 (27.5%)*	
					*Dialysis-specific DRPs*	*33 (26.5%)*	
					PhAR	81%	
					No. of interventions	102	
					*1 - adverse significance*	*0%*	
					*2 - no significance*	*6.9%*	
					*3 - somewhat significant *	*0%*	
					*4 - significant *	*78%*	
					*5 - very significant*	*4.9%*	
					*6 - extremely significant*	*1%*	
							
Possidente CJ et al. [12], (1999), HD and PD inpts	DS	37	3.5	CPS (MR, pts interviews, identification and resolution of DRPs)	No./Types of DRPs	161	
					*Pts did not receive drug*		
					*Overdosage*		
					*Labs needed*		
						More DRPs (77) at admission vs. discharge (41)	< 0.011
					PhAR	95.7%	
					Significance		
					*Somewhat significant*	*24.7%*	
					*Significant*	*58.4%*	
					*Very significant*	*16.9%*	
							
To LL et al. [26], (2001), HD outpts	BAS	49	6	Pharmacist-managed programmes compared to physician-managed pts	Mean HCT (± SD) during physician period vs. pharmacist period	35.36 ± 3.33/36.21 ± 3.46%	0.20
					Total EPO ? dose	8.5/7.7 million units	0.37
					Total elemental iron dose oral	85.605/95.550 mg	0.64
					Total elemental iron dose i.v.	13.600/33.025 mg	< 0.001
					Mean (± SD) Tsat level	29.82 ± 14.92/30.78 ± 13.17%	0.66
							
Viola RA et al. [27], (2002), HD outpts	DS	26	6	Pharmacist-managed hyperlipidaemia programmes with HD pts (laboratory management, counselling, statin initiation, and adjustments)	% of pts achieving LDL cholesterol target at baseline vs. Month 6	58%/88%	0.015
					Mean LDL (± SD) cholesterol at baseline vs. Month 6	96c5/80 ± 3 mg/dL	< 0.01
					Mean total cholesterol (± SD) at baseline vs. Month 6	170 ± 7/151 ± 4 mg/dL	< 0.01
					No./Types of interventions	15	
					*Dose increase*	*6*	
					*Drug change*	*5*	
					*Therapy initiation*	*2*	
							
Kimura T et al. [28], (2004), HD outpts	DS	41	9	Pharmacist-managed anaemia programmes	No. pts achieving the HCT target of >30% at baseline vs. Month 9	7 (17.1%)/32 (78%)	
					No. pts with EPO dose reductions due to intervention	23 (56%)	
							
Manley HJ et al. [29], (2004), HD outpts *Abstract*	DS	408	NR	Implementations of treatment algorithms for CV disease in HD pts by a pharmacist, collections of CV medication-related issues and recommendations to nephrologists, pts interview, MR	No. of recommendations	1575	
					PhAR	79.8%	
					Impact of recommendations on pts care		
					*Improvement*	*89.9%*	
					*No impact*	*7.6%*	
					*Worsened pts care*	*2.4%*	
					LDL cholesterol	-31.2 mg/dL	< 0.001
					HbA1C	-0.3%	NS
					Adjusted CV mortality hazard ratio	0.48 (CI 0.18, 1.3)	
							
Walton T et al. [30], (2005), HD outpts	DS	278	26	Pharmacist-managed anaemia programmes	Hb value at baseline and Month 6	9.5/11.8 g/dL	
					Mean (± SD) ferritin at baseline and Month 6	280.9 ± 326.4/431 ± 232.1 ng/mL	
					Mean (± SD) Tsat at baseline and Month 6	21 ± 7.9/33 ± 8%	
							
Sathvik BS et al. [32], (2007), HD outpts	RCT	90	4	Pharmacist-provided pts education	Medication knowledge (MKAQ) at baseline, Month 2 and 4 in Group 1 and 2	Improvement in MKAQ scores in Group 1 compared to baseline and to Group 2 at Month 2	< 0.05
				Group 1: Pharmacist pts education (Month 0-2)		No significant improvement in MKAQ scores in Group 2 compared to baseline at Month 2	>0.05
				Group 2: Usual health care w/o pharmacists (Month 0-2)		Improvement in MKAQ scores in Group 2 at Month 4 compared to baseline and to scores at Month 2	< 0.05
				Switch at Month 2		Decrease in MKAQ scores in Group I at Month 4 compared to Month 2	< 0.05
							
Erickson AI et al. [20], (2008), HD in- and outpts	DS	1184 pts visits	4	Prospective order review by CP and general CPS	Compliance with prospective order review	1059 (89.4%)	
					No./Types of interventions	77 (100%)	
					*Therapeutic-related*	*11 (14.3%)*	
					*Safety-related*	*49 (63.6%)*	
					*Compliance-related*	*17 (22.1%)*	
					PhAR	100%	
							
Castro R et al. [13], (2009), HD in- and outpts	BAS	60	6	MTM	Disease control parameters at baseline vs. follow-up visit at Day 90		
*Abstract*					SBP (MTM)	150 ± 22/144 ± 18 mmHg	0.12
					SBP (non-MTM)	143 ± 21/145 ± 25 mmHg	NS
					HbA_1c _(MTM)	9.2 ± 1.6/9.0 ± 2.0%	0.58
					HbA_1c _(non-MTM)	6.2 ± 1.2/6.5 ± 1.4%	NS
					Phosphorus (MTM)	6.2/5.6 mg/dL	.096
					Calcium/phosphorous product (MTM)	56 ± 19/50 ± 16	.03
							
Mirkov S [21], (2009), HD outpts	DS	64	8	DT reviews by CP	No./types of DRPs	278 (100%)	
					*Non-adherence*	*61 (22%)*	
					*Overdosage*	*26 (9.3%)*	
					*Untreated indication*	*24 (8.6%)*	
							
Pai AB et al. [22], (2009), HD outpts	RCT	104 (57/47)	24	INT: PC, DT reviews by CP	No./Types of DRPs	530 (100%)	
				CT: SOC, DT reviews by dialysis nurse	*Drug record discrepancy *	*133 (25%)*	
					*Untreated indication*	*111 (21%)*	
					*Subtherapeutic dosage*	*74 (14%)*	
					PhAR	100%	
					Reduction in drug use in INT	14%	< 0.05
					Reduction of hospitalisations in INT	42%	0.02
					Reduction of LOS in INT	21%	0.06
							
Pai AB et al. [23], (2009), HD outpts	RCT	107 (61/46)	24	INT: PC, DT reviews by CP	Total RQLP scores at Year 1 compared to baseline INT/CT	Worsening in Total RQLP score at Year 1 in CT group (88 ± 31/71 ± 34)	0.03
				CT: SOC, DT reviews by dialysis nurse	Total RQLP scores at Year 2 compared to baseline INT/CT	Improvement in INT/CT group, no statistically significant difference	

**BAS **before-after study, **CP **clinical pharmacist, **CPS **clinical pharmacy services, **CV **cardiovascular diseases, **DRP **drug-related problem, **DS **descriptive study, **DT **drug therapy, **EPO **erythropoietin, **HbA_1c _**glycosylated haemoglobin, **HCT **haematocrit, **HD **haemodialysis, **Hb **haemoglobin, **LDL **low-density lipoprotein, **LOS **length of stay, **MKAQ **medication knowledge assessment questionnaire, **MR **medication review, **MTM **medication therapy management service, **No**. number, **NR **not reported, **NS **not significant, **PC **pharmaceutical care, **PhAR **physician acceptance rate, **pts **patients, **RCT **randomised controlled trial, **RQLP **renal quality of life profile, **SBP **systolic blood pressure, **SD **standard deviation, **SOC **standard of care

^a ^Number of included patients in the intervention (INT) or control (CT) group

^b ^For brevity, only the three most commonly performed interventions/drug-related problems are listed.

### Scope of clinical pharmacy activities

The interventions performed in the included studies could be roughly grouped into the following categories: (1) general clinical pharmacy services (n = 12, 57.1%) [12-23], (2) clinical pharmacy services focusing on disease management (n = 7, 33.3%) [24-30] and (3) clinical pharmacy services with a focus on educational activities (n = 2, 9.5%) [31,32]. A listing of reported clinical pharmacist activities is provided in Table 4. The included studies report on the clinical pharmacists' involvement in the management of anaemia, lipid disorders, cardiovascular disease, hypertension, and diabetes in CKD or ESRD patients to various extents.

**Table 4:** Comprehensive listing of clinical pharmacy activities performed in CKD and ESRD patients

Medication review and monitoring of patient's pharmacotherapy regimen	Education and counselling	Disease management programmes	Further tasks
Taking a thorough medication history, including OTC drugs, herbal supplements, drugs prescribed by non-nephrologists, and CAM drugs	Provision of medical and therapeutic information for patients and other health care professionals	Basic clinical assessments during patient visists	Medication use evaluation
Medication review at different time points, such as at admission, during inhospital treatment, during each dialysis session, and at discharge	Training regarding the administration of drugs (e.g. ESAs self injections)	Ordering of laboratory tests	Audit measures
Matching computerised medication profiles with verbally obtained medication history	Counselling on side effects, interactions	Co-ordering of anaemia therapies and other drugs	
Medication order review and checking adherence to prescribing guidelines	Compiling of guidelines for proper drug use (e.g., iron and ESAs) and implementation of treatment algorithms (e.g., hyperlipidaemia, hypertension, and renoprotective drugs)	Co-prescribing within the scope of specific guidelines (e.g., anaemia management or lipid management)	
Development of discharge medication plans	Assessment and monitoring of compliance and adherence		
Identification of potential or actual DRPs			
Therapeutic recommendations (e.g. change of drugs, dose and/or interval adjustments, discontinuation of drugs, additional laboratory monitoring, nephrologist referral, addition of renoprotective drugs)			
Therapeutic monitoring (treatment, laboratory values, and specific drugs)			

### Outcomes

In 47.6% (n = 10) of the included studies [13,16,24-31], disease-oriented outcomes were reported, whereas patient-oriented outcome data were only available in four studies (19%) [22,23,31,32]. A synthesis of the disease- and patient-oriented outcome data is shown in Table 5. Four controlled trials (three of which were randomised) revealed that clinical pharmacy interventions had a positive impact on patient-oriented outcomes in the intervention group as compared to the available standard of care.

**Table 5:** Disease versus patient-oriented outcomes

Disease-oriented outcomes	Patient-oriented outcomes
Total cholesterol, LDL, HDL	Rate of hospitalization
HbA1c	Length of stay
Haematocrit, Tsat, ferritin, haemoglobin	Health-related quality of life
SBP, DBP	Medication-related knowledge
Phosphorus, calcium-phosphorus product	Renal quality of life
Drug dosages (e.g., EPO dosage or ferrous dosage)	Patient satisfaction survey

The third type of outcome parameter in the included studies was the total number of clinical pharmacist interventions performed or recommendations given together with the physician acceptance rate. These were considered primary (n = 7) or additional secondary (n = 3) outcome parameters in 10 out of 21 (47.6%) studies.

In the subanalysis of DSs, a weighted mean acceptance rate (± SD) based on study size of 78.7% (± 19.5) was calculated. DRPs were mainly classified according to the system presented by Strand et al. [33]. However, in several included studies, information on classification methodology was scarce, or a system developed by the authors was used. The DRPs most frequently described in the included studies were untreated indications, super- or supratherapeutic dosages and consequent dose adjustments, and medication record discrepancies. Assessments of the clinical significance of clinical pharmacist interventions were performed and reported in five of 10 included studies. For this purpose, the significance criteria published by Hatoum et al. [34] was used in two studies [12,19]. Unspecified categorisation systems were used in the other studies. Bias minimisation methods used during clinical significance assessments generally included a review by independent clinical pharmacists or the achievement of consensus among the ratings of clinicians, nephrologists and pharmacists.

Information on the drug classes among which the clinical pharmacists detected the majority of DRPs was reported in four of 10 studies [12,19,22,23]. The most commonly affected drugs were those used for treatment against renal bone disease and renal osteodystrophy together with anaemia and cardiovascular drugs.

The most common comorbidity in CKD or ESRD patients managed by clinical pharmacists was anaemia. Clinical pharmacists were primarily responsible for ordering and checking laboratory values and managing independent dosing and dose modifications of erythropoiesis-stimulating agents (ESAs) and iron within specific prescribing guidelines. Furthermore, comprehensive disease management programmes also included patient education and adherence-enhancing activities. Most of the studies [16,24,25,27-30] reported that a significantly higher proportion of patients managed by a clinical pharmacist maintained relevant target ranges (e.g., haemoglobin and haematocrit) as compared to patients receiving standard care. Aside from two studies addressing lipid management [27] and cardiovascular risk reduction in HD patients [29] through multiple disease interventions, no studies on diseases common to CKD or ESRD patients (e.g, hypertension or secondary hyperparathyroidism) or disease progression factors (e.g., proteinuria) with applicable inclusion criteria could be identified.

## Discussion

Our systematic review synthesises evidence on the impact of clinical pharmacist involvement in DRPs in general, with respect to different comorbidities (e.g., anaemia and lipid management), and regarding educational issues in CKD and ESRD patients.

Evidence of gaps in the care of patients with renal impairment (e.g., poor hypertension control, anaemia control in CKD and dialysis patients) is published in the literature [8,9]. For the patient's sake, these gaps must be addressed using all available methods. Enhancing the involvement of clinical pharmacists may be one potential strategy. Thus, for example, clinical pharmacist-led programmes showed higher proportions of CKD patients achieving haemoglobin target [25], increased medication knowledge [32], decreased hospitalisation rates [22], and an overall improvement in the quality of life of dialysis patients [23].

By addressing the issues illustrated in Table 4 in their general and more specified clinical work, clinical pharmacists fulfil the requirements stated in the NKF-KDOQI Guidelines "Chronic Kidney Disease: Evaluation, Classification and Stratification" [35], which explicitly highlight the need for regular medication reviews, including dosage adjustment, adverse drug event (ADE) detection, drug interaction detection, and therapeutic drug monitoring (TDM). Given the nature of their major responsibilities and tasks, clinical pharmacists interact with patients, physicians, and other health professionals and share the goal of optimising pharmacotherapy and patient care. This multidisciplinary and multilevel approach is underlined by all included studies. Clinical pharmacists, as pharmacotherapy experts, are engaged in the care of the CKD and ESRD patient population at different stages. Potential responsibilities of clinical pharmacists may comprise attainment of blood pressure, glycaemic, and lipid goals, and the early evaluation and treatment for proteinuria, anaemia, and secondary hyperparathyroidism, among others [10]. Optimal control of hyperglycaemia, including maximal suppression of urinary albumin excretion by angiotensin-converting enzyme inhibitors (ACEIs) or angiotensin II receptor blockers (ARBs) in diabetic patients with persistent microalbuminuria, and hypertension can limit progression of CKD to ESRD. Evidence of improved glycaemic and blood pressure control and decreased levels of microalbuminuria through clinical pharmacists' involvement in patient care is published [36-38]. However, due to our search strategy, studies explicitly addressing only these latter aspects are not included in this systematic review. Furthermore, no study of clinical pharmacy services in CKD patients investigating the slowing down of disease progression could be identified.

The CKD and ESRD population can be characterised by its vulnerability and susceptibility to drug-therapy-related morbidity due to many factors. Commonly reported DRPs in CKD or ESRD patients (e.g., dosing problems and medical record discrepancies) are not surprising given the complexity of dosing during either type of renal replacement therapy due to common changes in drug pharmacodynamics and pharmacokinetics [39]. This fact is further aggravated by the high number of concomitant drugs used and comorbidities, as studies report an average number of 10 to 12 drugs per day and five comorbidities for HD patients [7]. Intensified care and additional monitoring are warranted for patients taking more than five drugs, patients with more than 12 total medication doses, patients with drug regimens prone to frequent changes and three or more concurrent disease states, and patients with a history of non-compliance, and the presence of drugs requiring TDM [40]. CKD and HD patients generally fulfil all of these criteria and therefore warrant increased monitoring. Problems with medical record discrepancies and the accuracy of medication profiles, which are among the most commonly reported DRPs, are further highlighted in a prospective observational study of 63 HD patients, which reports record discrepancies in 60% of all patients. Several clinical pharmacy studies provide insights into the risk factors for DRPs. One study [15] highlights an inverse correlation between residual kidney function (based on creatinine clearance) and the number of DRPs. Another study reports a positive correlation between the number of DRPs, on the one hand, and age and length of time on dialysis, on the other [21]. All of these aspects present opportunities for clinical pharmacist to engage in CKD and ESRD patient care.

Generally, more than three-quarters of clinical pharmacist interventions and suggestions were accepted by physicians. This physician acceptance rate is well within the range of other reported acceptance rates based on a review of clinical pharmacist impact on DRPs and clinical outcomes [6]. Due to the use of different classification systems and the resulting heterogeneity of DRPs, a profound statistical analysis was not performed.

No studies could be identified that explicitly addressed the issue of adherence in CKD or ESRD patients; nonetheless, it presents a major barrier to optimal patient care. Especially among patients taking a high number of prescription drugs, complex medication schemes and long treatment periods cause adherence to wane [41]. Guaranteeing a high level of medication knowledge may be one strategy to increase adherence and to prevent DRPs resulting from incorrect drug use or overall failure to take medications. Clinical pharmacist intervention to improve patient medication knowledge was the study objective in two of the included studies [31,32], which could be achieved.

We identified a higher proportion of studies investigating disease-oriented versus patient-oriented outcomes. Patient-oriented outcomes are those that directly matter to patients, that is, those regarding longer life and improved quality of life. From an evidence-based point of view, studies investigating patient-oriented outcomes contribute more to the overall evidence and therefore have to be weighted more heavily. However, further studies with hard endpoints, as highlighted in Table 5 as well as longer study periods are definitely warranted, as they provide further evidence on the role of pharmacists in the care of CKD and ESRD patients and other patient groups.

Several studies on clinical pharmacist involvement were identified by our search strategy, but only four of them were controlled trials (two by the same authors) with high-quality methodological design and therefore a higher evidence impact. We decided to also include abstracts in our review, because we are convinced that these small studies of the impact of clinical pharmacy on patient care contribute to the overall evidence on this topic. We could not identify any studies that specifically addressed PD patients. However, in the two studies that included PD patients, the authors did not comment on any special issues (e.g., regarding the type of DRPs or adherence). Given the complexity and specifics of drug dosing during PD, the high need for education and patient training, and the high risk of infections (e.g., peritonitis), data specific to this patient population would be interesting and warranted. We hypothesise that clinical pharmacists are routinely integrated into different aspects of PD patient care, but due to irregular clinical attachment (as compared to HD patients, who generally attend clinic three times per week), such studies are more difficult to perform.

Furthermore, regarding CKD patient studies, it was not possible to subdivide different clinical pharmacist activities and further relevant findings (e.g., common DRPs and performed interventions) according to CKD stage.

Our review is subject to publication bias. We could not identify any studies showing that clinical pharmacy interventions had a negative impact on patient care. Furthermore, studies that used DRPs and physician acceptance rates as outcome parameters lacked information about rejected interventions and the reasons for rejection. The reporting of clinical significance assessments for performed interventions increases the scientific value of clinical pharmacy research, primarily by reducing bias. Data on the impact of clinical pharmacists on hospitalised inpatients is also scarce. In addition, the majority of the studies were published in the United States. Interestingly, only one study [31] from Europe could be identified; three of the remaining four studies were from Asia [14,28,32], and one was from New Zealand [21]. However, we hypothesise that clinical pharmacists are widely engaged in the care of CKD and ESRD patients. There are, for example, special interest groups dedicated to their care, such as the United Kingdom Renal Pharmacist Group [42]. Further high-quality studies on the impact of clinical pharmacists on key issues such as adherence and disease progression are thus warranted.

## Conclusions

All identified studies on the involvement of clinical pharmacists in the care of CKD and ESRD patients showed some benefit. However, high-quality evidence on the impact of clinical pharmacy services is limited to a few studies. Clinical pharmacists address areas requiring improvement as well as unmet DRPs responsively and preventatively. By doing so, clinical pharmacists positively contribute to the care of patients with impaired renal function and reduce the gaps in current patient care.

## List of abbreviations used

ACCP: American College of Clinical Pharmacy; ACEI: angiotensin-converting enzyme inhibitor; ARB: angiotensin II receptor blocker; ADE: adverse drug event; BAS: before-after study; CAM: complementary and alternative medicines; CHD: coronary heart disease; CKD: chronic kidney disease; CVD: cardiovascular disease; DS: descriptive study; DRP: drug-related problem; DTP: drug-therapy problem; EKHA: European Kidney Health Alliance; ESA: erythropoiesis stimulating agent; ESRD: end-stage renal disease; HD: haemodialysis; IPA: International Pharmaceutical Abstracts database; RCT: randomised controlled trial; RRT: renal replacement therapy; PD: peritoneal dialysis; TDM: therapeutic drug monitoring.

## Competing interests

The study was performed within a clinical pharmacy project that was funded by Amgen. The authors declare that there are no financial or other conflicts of interests with respect to the contents of the article.

## Authors' contributions

GS was responsible for the study design, data collection and interpretation and preparation of the manuscript. RLG was responsible for the study design, data interpretation and review of the manuscript. All authors read and approved the final manuscript.

## Pre-publication history

The pre-publication history for this paper can be accessed here:

http://www.biomedcentral.com/1471-2369/12/35/prepub
